# miR-651-3p Enhances the Sensitivity of Hepatocellular Carcinoma to Cisplatin via Targeting ATG3-Mediated Cell Autophagy

**DOI:** 10.1155/2021/5391977

**Published:** 2021-08-18

**Authors:** Lei Zou, Peng Sun, Lei Zhang

**Affiliations:** Department of Gastroenterology, Zibo Central Hospital, Zibo 255036, Shandong Province, China

## Abstract

Drug resistance is a major challenge for hepatocellular carcinoma (HCC) treatment in a clinic, which limits the therapeutic effect of the chemotherapeutic drugs, including cisplatin (CDDP), in this disease. Mounting evidence has identified that miRNAs dysfunction is related to the resistance of tumor cells to CDDP, and miR-651-3p has been identified as a tumor inhibitor to suppress the progression of multiple tumors. However, the role of miR-651-3p in HCC remains unclear. In this study, the relative expression of miR-651-3p in HCC tissues and cell lines were measured, and the functions of miR-651-3p were also observed by CCK-8 assay, flow cytometry assay, and Western blot. Moreover, the downstream target of miR-651-3p was predicted and verified via TargetScan and dual-luciferase reporter assay, and its functions were also investigated. The results showed that miR-651-3p was significantly downregulated in HCC tissues and cell lines, and the decreased miR-651-3p was also observed in CDDP-induced cells. miR-651-3p upregulation could effectively inhibit the proliferation and induce the apoptosis of R-HepG2. It was also found that ATG3 was a downstream target of miR-651-3p, and ATG3 was highly upregulated in HCC tissues. Moreover, the upregulated ATG3 could partly reverse the effects of miR-651-3p on R-HepG2. Besides, miR-651-3p involved the autophagy pathway of the HCC cells via targeting ATG3. In conclusion, miR-651-3p could regulate the autophagy to enhance the sensitivity of HepG2 cells to CDDP via targeting ATG3.

## 1. Introduction

Hepatocellular carcinoma (HCC) is the fifth most common malignant disease in the world, which is also the major leading cause of cancer-related death [1]. Statistically, more than 60% of patients have advanced HCC at the time of diagnosis [2]. Several chemotherapy drugs approved by the Food and Drug Administration (FDA) have shown high efficacy in controlling the symptoms of the patients in the early stage, while their therapeutic effects are reducing significantly with the decrease of the sensitivity of tumor cells to the drugs [3, 4]. Chemotherapy drugs involved some special targets in tumor cells, and tumor cells can develop a serious mechanism to weaken the injury and escape the apoptosis induced by drugs, which is a major cause leading to the failure of chemotherapy in the long-term intervention [5, 6]. Autophagy, a natural survival mechanism formed in cell involution, protects cells from apoptosis induced by injury of toxicants, oxidative stress, and physical damages [7, 8]. The activated autophagy has been observed in multiple tumor cells, and some studies have indicated that activated autophagy effectively suppresses the malignant progressions of tumor cells in the early stage [9]. However, accumulating evidence has indicated that autophagy may decrease the drug's toxicity in cells and minimize their therapeutic effect on tumor cells.

MiRNAs consisted of 18–22 nucleotides and play essential roles in cells activities [10]. MiRNAs dysfunction involves proliferation and invasion, and increasing studies have confirmed that some miRNAs are involved in the regulation of HCC cells to chemotherapeutical drugs [11]. For instance, miR-124 downregulation is related to the stem formation and sorafenib resistance of HCC cells [12]. A previous study has identified miR-651-3p as a tumor inhibitor that can effectively suppress the progression of glioma cancer [13]. However, the role of miR-651-3p in HCC remains unclear. This study focused on investigating the connection of miR-651-3p and HCC and intended to illustrate the regulation mechanism of miR-651-3p on HCC.

This study attempted to investigate the role of miR-651-3p in HCC and provide a potential basis for HCC treatment.

## 2. Materials and Methods

### 2.1. Pathological Tissue

The tumor tissues and normal tissues of the 15 patients at ZiBo Central Hospital, Zibo, Shandong, China, were collected for this investigation. All tissues were frozen at −80°C after sample collection. The study was approved by the Ethics Committee of the ZiBo Central Hospital, Zibo, Shandong, China, and all patients provided informed written consent.

### 2.2. Cell Culture

The HCC cells lines, including HepG2 and Huh7, purchased from (BioWit Technologies Co., Ltd., Shenzhen, China) were used for this study. All cells were cultured with Dulbecco's modified Eagle's medium containing 10% fetal calf serum (DMEM+10% FBS, BioWit Technologies Co., Ltd., Shenzhen, China). The cells were cultured in an incubator with 5% CO_2_ and 37°C.

The expression vectors of ATG3, miR-651-3p mimics, and their related negative controls were purchased from Generay Biotech (Shanghai, China). The cells were seeded in 6-well plates and cultured at the incubator with 37°C and 5% CO_2_, and the cell transfection was performed when cells confluence at 70%. In brief, 100 pmol of RNA or 4 *μ*g of DNA was incubated with 250 *μ*L serum-free medium for 5 min, and 10 *μ*L of Lipofectamine 2000 (Generay Biotech, Shanghai, China) was also diluted with the 250 *μ*L serum-free medium. After that, the medium containing the expression vectors of ATG3 or miR-651-3p mimics was incubated with isometric-diluted Lipofectamine 2000 at 25°C for 20 min. Finally, 500 *μ*L of the mixtures was added to each well, and then, the cells were cultured for 24 hours.

### 2.3. qRT-PCR

The total RNAs were extracted from the tissues and cells with TRIzol reagent, and the concentrations of the extracts were measured by ultraviolet spectrophotometry. 1 *μ*g of RNAs was reversed as cDNA with TaqMan MicroRNA Reverse Transcription Kit (Applied Biosystems, Foster City, CA). The primers were synthesized and purified by RiboBio (Guangzhou, China). 10 *μ*L of reaction system was prepared according to the instructions of the KAPA qRT-PCR kit (Sigma-Aldrich, Missouri, USA). The reaction conditions included the denaturation at 95°C for 3 min, followed by amplification for 40 cycles at 95°C for 12 s, 56°C for 40 s, and 70°C for 30 s. The relative expression levels of miR-651-3p were calculated with the 2^−(ΔΔ*Ct*)^ method. The primers of miR-651-3p, ATG3, and U6 are listed in Table 1.

### 2.4. Cell Viability Assay

The viability of the cells was observed by CCK-8 assay. 5 × 10^4^ cells were seeded into the 96-well plates. After cell transfection, the viability of the cells at 0, 24, 48, and 72 hours were measured by CCK-8 assay. Briefly, 10 *μ*L of CCK-8 solution (Solarbio Biotechnology Co., Ltd., Shanghai, China) was added into each well, and then, the cells were further cultured in the dark for 4 hours. The viability of the cells was observed by a microplate reader (Molecular devices, Shanghai, China) at 450 nm.

### 2.5. Flow Cytometry Assay

Flow cytometry assay was performed to observe the effects of miR-651-3p on the apoptosis of HCC cells. 2 × 10^3^ cells were suspended by 1 mL of ice Annexin V-FITC binding buffer, and then, 10 *µ*L of propidium iodide (PI 20 *µ*g/ml, Shanghai Yuanye Biotechnology Co., Ltd, Shanghai, China) and 5 *µ*l of Annexin V-FITC (10 *µ*g/ml, Shanghai Yuanye Biotechnology Co., Ltd, Shanghai, China) were added into the cells. After incubation for 15 min, the apoptosis level of the cells was observed by flow cytometry equipment (BD Biosciences, State of New Jersey, USA).

### 2.6. Western Blot

Total proteins were extracted from the tissues and cell lines with RIPA buffer, and BCA kit (ThermoFisher, Massachusetts, USA) and UV spectrophotometry were used to measure the concentration of the proteins. The proteins were boiled for 5 mins, and then, they were separated with sodium dodecyl sulfate polyacrylamide gels. Subsequently, the proteins in gels were translated on the polyvinylidene fluoride membranes (BioRad, CA, USA) with a wet translation method. After that, the membranes were blocked with 5% fat-free milk, and the related primary antibodies including anti-ATG3(1 : 1000, ab2577009 ThermoFisher, Massachusetts, USA), anti-LC3B (1 : 1000, ab2234770, ThermoFisher, Massachusetts, USA), and anti-*β*-actin (1 : 1000, sc-47,778, Santa Cruz) were used for membrane incubation at 4°C overnight. The membranes were washed with TBST for three times (15 min/time), and then, they were incubated with second antibodies for 2 hours. After membranes washing (3 × 10 min), the chemiluminescence detection system was used to observe and calculate the relative expressions of the related proteins.

### 2.7. Statistical Analysis

All experiments in this study were performed three times, independently. The data were analyzed with SPSS 20.0 and expressed by GraphPad Prism 8. The difference between the two groups was analyzed and calculated with the chi-squared test or ANOVA (Tukey's post hoc test). *P* < 0.05 means that the difference was statistically significant.

## 3. Results

### 3.1. miR-651-3p Was Downregulated in HCC Tissues and Cells

**Figure 1:**
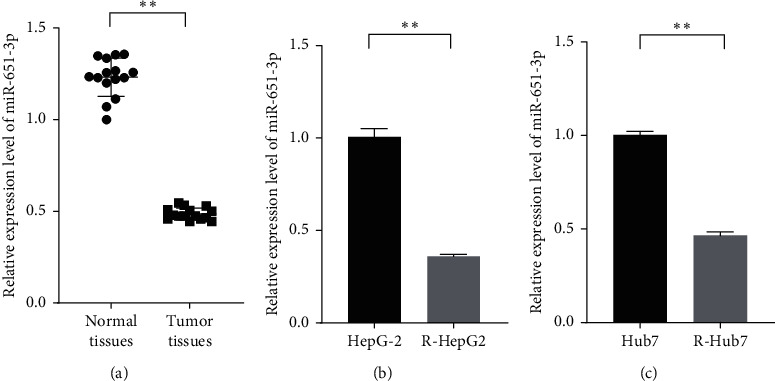
miR-651-3p was downregulated in HCC tissues and drug-resistant HCC cells. (a) The relative expression levels of miR-651-3p in normal and tumor tissues measured by qRT-PCR. (b) The expression levels of miR-651-3p in HepG2 cells and resistant HepG2 (R-HepG2) were measured by qRT-PCR; (c) The expression levels of miR-651-3p in Hub7 cells and resistant Hub7 (R-Hub7) measured by qRT-PCR.

To investigate the involvement of miR-651-3p in HCC, the qRT-PCR was used to measure the expression levels of miR-651-3p in HCC tissues and cell lines. The results showed that miR-651-3p was highly downregulated in the HCC tissues compared with the paracancerous tissues of the patients. Subsequently, the expression level of miR-651-3p was also measured in CDDP-sensitive and CDDP-resistant tissues. The results showed that miR-651-3p was significantly downregulated in CDDP-resistance tissues (Figure 1(a), *p* < 0.01). Moreover, the miR-651-3p levels were also investigated in CDDP-resistant HepG2 cells (R-HepG2) and CDDP-resistant Huh7 (R-Huh7). It was found that miR-651-3p was significantly downregulated in R-HepG2 cells (Figure 1(b), *p* < 0.01). Those observations suggested that decreased miR-651-3p was involved in the resistance of HepG2 cells.

### 3.2. miR-651-3p Enhanced the Sensitivity of R-HepG2 on CDDP

**Figure 2:**
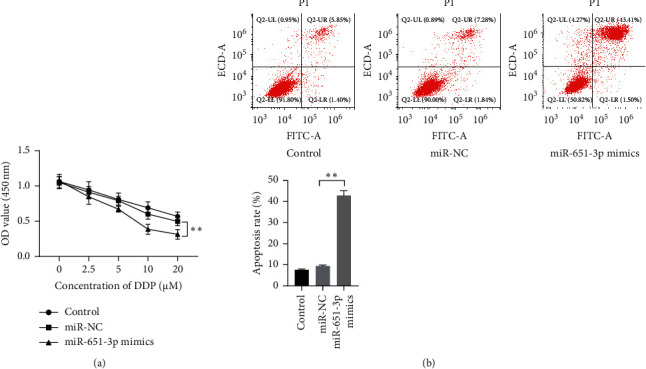
miR-651-3p increased the sensitivity of R-HepG2 cells on CDDP. (a) The viability of the R-HepG2 after serial dosage DDP treatment observed by CCK-8. (b) The apoptosis level of R-HepG2 with 5 *μ*M of CDDP measured by flow cytometry assay.

To evaluate the function of miR-651-3p in resistance of HepG2, the miR-651-3p mimics and related miR-NC were, respectively, transfected into R-HepG2 cells, and the cells were cultured with the medium containing a different dose of CDDP range from 0 *μ*M to 50 *μ*g/mL. The CCK-8 and cytometry assay were used to observe the viability and apoptosis of the cells with 5 *μ*M of CDDP. The results showed that the viability levels of R-HepG2 transfected with miR-NC in different doses were significantly high compared with the cells transfected with miR-651-3p (Figure 2(a), *p* < 0.01). Moreover, the flow cytometry assay showed that HepG2 cells with the high miR-651-3p level expressed a high apoptosis rate compared with the cells with the low miR-651-3p level (Figure 2(b), *p* < 0.01). Those observations suggested that miR-651-3p could effectively enhance the resistance of R-HepG2 on CDDP.

### 3.3. miR-651-3p Directly Targets ATG3, and ATG3 Involved Resistance of HepG2

**Figure 3:**
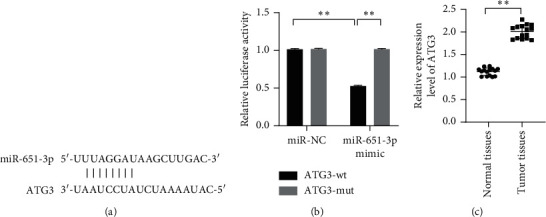
ATG3 was a downstream target of miR-651-3p and was significantly upregulated in tumor tissues. (a) The binding sites of miR-651-3p and ATG3. (b) The binding effect of miR-651-3p and ATG3 observed by dual-luciferase reporter assay. (c) The mRNAs level of miR-651-3p significantly upregulated in tumor tissues.

To explore the downstream target of miR-651-3p, MiRDB was used to search the downstream target of miR-651-3p. The results showed that the 3′-UTR of ATG3 could match with the sequence of miR-651-3p (Figure 3(a), *p* < 0.01). Subsequently, the miR-651-3p mimics, miR-NC, ATG3-wt, and ATG3-mut were, respectively, transfected into the HEK-293T cells to verify the binding effects of miR-651-3p and ATG3. It was found that miR-651-3p remarkably suppressed the activity of ATG3-wt, while it did not express any significant effect on ATG3-mut (Figure 3(b), *p* < 0.01). Besides, the expression levels of ATG3 were also measured in HepG2 and R-HepG2 cells. The results showed that ATG3 was significantly upregulated in R-HepG2 cells compared with HepG2 cells (Figure 3(c), *p* < 0.01). Those observations suggested that ATG3 could be targeted by miR-651-3p and involved the chemotherapeutic resistance of HepG2 cells.

### 3.4. ATG3 Reversed the Effects of miR-651-3p on R-HepG2

**Figure 4:**
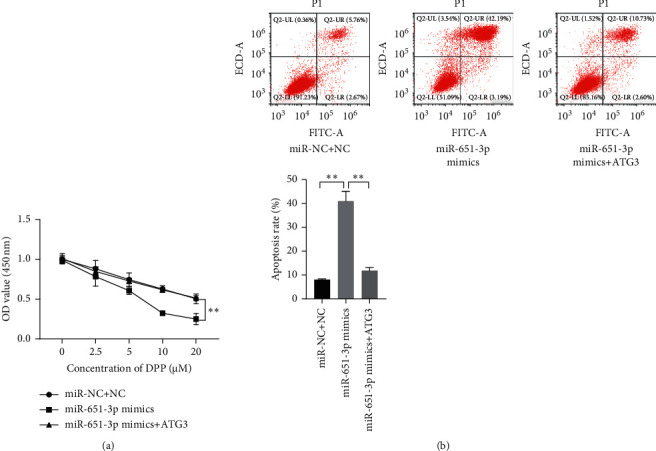
ATG3 reversed the effect of miR-651-3p on the CDDP-resistance of R-HepG2 cells. (a) The viability of the resistant HepG2 after serial dosage DDP treatment observed by CCK-8. (b) The apoptosis level of resistant HepG2 with 5 *μ*M of CDDP measured by flow cytometry assay.

Although it was confirmed that ATG3 was significantly upregulated in R-HepG2 cells, whether ATG3 plays a key role in the regulation of miR-651-3p on R-HepG2 remains unknown. The cells were cultured with the medium containing 5 *μ*M of CDDP, and the miR-651-3p mimics and ATG3 expressed vectors were cotransfected into the R-HepG2 to observe the changes in viability and apoptosis levels. CCK-8 assay showed that the enhanced sensitivity of R-HepG2 induced by miR-651-3p upregulation could be reversed by ATG3 (Figure 4(a), *p* < 0.01). The flow cytometry assay showed that ATG3 could effectively inhibit the apoptosis of R-HepG2 with a high miR-651-3p level (Figure 4(b), *p* < 0.01). Those observations suggested that miR-651-3p could elevate the sensitivity of R-HepG2 on CDDP.

### 3.5. miR-651-3p Regulated Autophagy Level of R-HepG2 via Targeting ATG3

**Figure 5:**
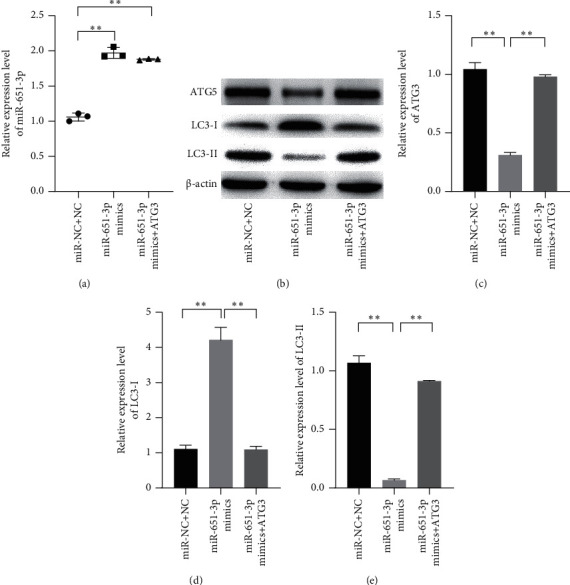
miR-651-3p regulating the cell autophagy of R-HepG2 via targeting ATG3. (a) The relative expression level of miR-651-3p measured by qRT-PCR. (b–e) The relative expression levels of ATG3, LC3-I, and LC3-II measured by Western blot, respectively.

To illustrate the regulation mechanism of miR-651-3p in resistance of HCC, the related proteins of the autophagy pathway in R-HepG2 cells were measured by Western blot after transfecting with miR-651-3p mimics and ATG3 vectors. The result showed that the miR-651-3p significantly increased LC3-I and reduced LC3-II in R-HepG2 cells. However, the effects of miR-651-3p on the cells could be reversed by ATG3. Besides, it was also found that increased miR-339-5p significantly inhibited the expression of p-AMPK in R-HepG2 cells (Figure 5, *p* < 0.01). Those observations suggested that miR-651-3p inhibited the AMPK-induced cell autophagy via targeting ATG3.

## 4. Discussion

Remarkable progression has been made in HCC treatment in recent ten years [14]. However, completely healing HCC remains quite difficult. At present, chemotherapy failure induced by drug resistance has become a major challenge in the clinical field of HCC treatment [15]. This study investigated the role of miR-651-3p in HCC and revealed the regulation mechanism of miR-651-3p involved resistance in HCC to CDDP.

MiRNAs dysfunction has been confirmed as a major reason for cancer development. Increasing studies have also indicated that the malignant progression of HCC is related to the miRNAs dysfunctions [16]. In this study, it was found that miR-651-3p was obviously downregulated in HCC tissues and cell lines. miR-651-5p locates on the chromosome 3, p13 of humans, and the miR-651-5p involves the artery remodeling. The reduced miR-651-3p in gastric cancer cells has been observed by a recent study, while the role of miR-651-3p in other tumors remains unclear [17]. This study proved that miR-651-3p downregulation was related to the resistance of HCC cells to CDDP. Decreased miR-651-3p has been found in gastric cancer, and the proliferation and migration of the tumor cells can be effectively inhibited when miR-651-3p is overexpressed [13]. Therefore, it was hypothesized in this study that miR-651-3p involved the progression of HCC. In this study, it was also observed that miR-651-3p was highly downregulated in CDDP-resistant HepG2 cells. CDDP is one of the effective drugs for cancer intervention, which has been widely used in cancer treatment [18, 19]. Moreover, the study also found that miR-651-3p upregulation could effectively reduce the viability and promote the apoptosis of R-HepG2 cells. Thus, this study supports that miR-651-3p deficiency is a major reason for the resistance of HepG2 on CDDP.

MiRNAs are characterized by regulating the expression of proteins to involve the progressions of cells via targeting the 3′-UTR of the related mRNAs [20]. Given the characters of miRNAs in regulating the expression of proteins, the downstream target of miR-651-3p was searched by TargetScan. It was found that ATG3 was a target of miR-651-3p. ATG3 has been found as a tumor promoter that involves the malignant behaviors of multiple tumors [21]. The promotion of ATG3 on tumor cells has been confirmed by several studies, and ATG3 was regulated by multiple miRNAs to involve the malignant progression of some tumor cells [22, 23]. Moreover, ATG3 is also related to the sensibility of the cell on DNA injury [24].

Several studies have proved that ATG3 was significantly upregulated in multiple cancers, and ATG3 downregulation could effectively inhibit the proliferation invasive abilities of tumor cells [25]. Huang et al. have found that ATG3 downregulation significantly inhibited the proliferation, migration, and invasion of nonsmall cell lung cancer [26]. In this study, it was proved that ATG3 restoration could partly rescue the enhanced sensitivity of R-HCC cells to CDDP induced by miR-651-3p and reduce the apoptosis rate of the cells when suffered CDDP treatment. Thus, this study suggested that ATG3 involved the regulation of miR-651-3p on the resistance of HCC. Drug-resistance is related to the autophagy progression of the cells, and tumor cells can reduce the effect drug toxicity of chemotherapy drugs via promoting cell autophagy [27]. ATG3 is related to cell autophagy, and several studies have found that aberrant expression of ATG3 can increase cell autophagy, which is related to enhanced drug-resistance of tumor cells [28]. In this study, the expression of the related proteins in the autophagy pathway was observed, and the obvious changes in the expression levels of LC3-I and LC3-II were observed. Thus, this study supported that miR-651-3p could inhibit the ATG3-mediated cell autophagy to enhance the sensitivity of HCC cells to CDDP.

This study revealed miR-651-3p as a tumor inhibitor in HCC cells and illustrated that miR-651-3p could enhance the sensitivity of HCC cells on CDDP via regulating the aberrant autophagy levels of HCC cells induced by increased ATG3. However, the presumption in this study was only confirmed with cell experiments, and more evidence from animal experiments is still necessary to validate the resistance-inhibited effects of miR-651-3p on HCC.

## Figures and Tables

**Table 1:** Primer sequence of miR-493-5p, E2F3, and U6.

Name of primer	Sequences

miR-493-5p-F	5′-GCGCAAAGGAAAGTGTATCC-3′
miR-493-5p-R	5′-CAGTGCGTGTCGTGGAGT-3′
ATG3-F	5ʹ-GCAAACAAGAACCTATGACCTG-3
ATG3-R	5ʹ-GTCTTCATACATGTGCTCAACTG-3ʹ
U6-F	5′-CTCGCTTCGGCAGCACA-3′
U6-R	5′-AACGCTTCACGAATTTGCGT-3′

## Data Availability

The data used to support the findings of this study are available from the corresponding author upon request.
